# Targeted radioimmunotherapy with the iodine-131-labeled caerin 1.1 peptide for human anaplastic thyroid cancer in nude mice

**DOI:** 10.1007/s12149-021-01618-3

**Published:** 2021-05-04

**Authors:** Ruoting Lin, Bowei Ma, Na Liu, Lu Zhang, Tiantian He, Xiongying Liu, Tongsheng Chen, Wenjuan Liu, Yongnan Liang, Tianfang Wang, Guoying Ni, Xiaosong Liu, Ning Yang, Jinhe Zhang, Jianwei Yuan

**Affiliations:** 1 Department of Nuclear Medicine, The First Affiliated Hospital/Clinical Medical School, Guangdong Pharmaceutical University, Guangzhou, 510080 Guangdong China; 2 Department of TCM Resident Training, The First Affiliated Hospital of Guangzhou University of Traditional Chinese Medicine, Guangzhou, 510405 Guangdong China; 3 The First People’s Hospital of Foshan, Foshan, 528000 Guangdong China; 4 Genecology Research Centre, University of Sunshine Coast, Maroochydore DC, QLD 4558 Australia; 5 Department of Nuclear Medicine, General Hospital of the Southern Theatre Command, People’s Liberation Army of China, Guangzhou, 510010 Guangdong China

**Keywords:** Radioimmunotherapy, Caerin 1.1 peptide, Iodine-131, Anaplastic thyroid cancer, CAL-62 cell

## Abstract

**Objective:**

The combination of two or more drugs with different mechanisms is a promising strategy for cancer treatment, and radioimmunotherapy (RIT) is a trending antitumor strategy. Radiotherapy (RT) can promote and activate antitumor immune effects, and immunotherapy can strengthen the effects of selective internal radiotherapy (SIRT); the RIT combination is synergistic and can overcome the adverse side effects of monotherapy. In this study, we developed a radioimmunoconjugate (RIC)—the iodine-131 (^131^I)-labeled caerin 1.1 peptide—to treat human anaplastic thyroid cancer (ATC).

**Methods:**

Antitumor activity of caerin 1.1 peptide was determined by MTT assay, plate colony formation and cell wound scratch assays, and the mechanism of the inhibition of carein 1.1 peptide on the growth of CAL-62 cells was identified by cell cycle and western blot. Then, we investigated the efficacy of the caerin 1.1 peptide as a single drug and the ^131^I-labeled caerin 1.1 peptide for ATC. H&E and TUNEL staining was performed to detect dead cells in the tumor tissue sections.

**Results:**

We found that caerin 1.1 arrested cells in the S phase to induce apoptosis and inhibited tumor growth to inhibit phosphorylation of Akt. In vivo, the iodine-131 (^131^I)-labeled caerin 1.1 peptide achieved better antitumor efficacy than radiotherapy alone and showed a good biosafety profile.

**Conclusions:**

Our study demonstrates for the first time that the iodine-131 (^131^I)-labeled caerin 1.1 peptide can inhibit CAL-62 tumor growth and migration. The iodine-131 (^131^I)-labeled caerin 1.1 peptide, which represents a radioimmunotherapy strategy based on the combination of SIRT with a peptide–drug conjugate, could provide a treatment means for the radical cure of ATC.

## Introduction

Radioimmunotherapy (RIT) is an effective antitumor strategy for coupling radioisotopes to specific molecules that identifiably and selectively seek the cancer cells, one form of which is selective internal radiotherapy (SIRT), radiation can be preferentially directed to disease sites [1, 2]. Radiotherapy (RT) is widely used in clinic as a radical treatment for local cancer or isolated metastasis, more than 50% of cancer patients receive RT to combat tumor progression [3]. However, some high-grade malignancies are resistant to ionizing radiation [4]. Compared with other treatments, the major advantage of RIT is the direct delivery of radiopharmaceuticals into the tumor tissue, which maximizes the antineoplastic effects on tumor tissue while protecting normal tissues from radiation damage [5]. The application of radioimmunoconjugates (RICs) in the brachytherapy of malignant tumors by vascular intervention or by direct implantation has achieved remarkable success, over the past 25 years, a wide variety of RICs labeled with radioisotopes (i.e., ^111^In, ^89^Zr and ^131^I) have been explored and translated to the clinic [6–8]. Among the numerous radionuclides used in diagnosis and therapy, iodine-131 (^131^I) is a rare radioisotope with both γ- and β-particle emission characteristics; it has a half-life of 8.1 days and is routinely commonly used in radionuclide labeling [9]. Moreover, free ^131^I that dissociates from a RICs accumulates in the thyroid gland or is excreted, but is not excreted from the body but accumulates in the liver, spleen, and bone, which has almost no side effects [10]. Therefore, ^131^I has gained much attention and ^131^I-labeled RICs have been applied for various kinds of tumor theranostics [10–13]. Immunotherapy is becoming one of the important methods of cancer treatment, and the monitoring mechanism of the tumor cell immune escape system is being revealed [14]. More than 200 host-defense peptides have been isolated and identified from the skin secretions of Australian frogs or toads in the past 2 decades. Among them, the caerin peptide can inhibit cancer cell behaviors by executing the TNF-α signaling pathway and can interfere with the tumor microenvironment (TME) by recruiting antigen-specific T cells to tumor sites [15–17].

Immunotherapy has achieved significant benefits in some cancer patients, but many patients still do not benefit from these drugs in terms of clinical outcomes and durable responses [14, 18, 19]. Therefore, additional interventions are needed to induce an effective response to immunotherapy. RT has been considered an immunosuppressive treatment, and it has been extensively reported to have immunomodulatory effects in preclinical and clinical studies [2, 20]. RT releases tumor-specific antigens and activates the immune signaling pathway [21], accelerates programmed cell death by immune mediation [22], modulates the TME, increases immunocyte infiltration in tumors [23], stimulates tumor-specific cytotoxic T cells, and enhances T cell homing, engraftment and function in tumors [9, 24]. Therefore, the synergistic effect of RT and immunotherapy is well known. Several studies have reported that RIT has become an effective type of cancer treatment. The United States Food and Drug Administration (US FDA) approved two RICs, Bexxar^®^ (^131^I-tositumomab) [25] and Zevalin^®^ (^90^Yibritumomab tiuxetan) [26], in the early 2000s as first-line treatments for relapsed or refractory non-Hodgkin lymphoma [27].

Our previous work demonstrated that the caerin peptide is able to be labelled with ^131^I for targeted therapy of tumors in vitro [28]. Herein, we investigated the efficacy of the caerin 1.1 peptide as a single drug and the ^131^I-labeled caerin 1.1 peptide as a RIC for ATC. We also assessed the fundamental mechanism of the antitumor activity of the caerin 1.1 peptide. In addition, we developed a reliable mouse model of ATC and investigated whether the ^131^I-labeled caerin 1.1 peptide RIC in vivo could enhance the antitumor effect. Our results showed that the ^131^I-labeled caerin 1.1 peptide has potential in tumor-targeted RIT.

## Materials and methods

### Mice

We purchased 6–8-week-old adult female BALB/C nude mice that were specific pathogen free (SPF) from Guangdong Medical Laboratory Animal Center and raised them at the Animal Resource Centre (First Affiliated Hospital of Guangdong Pharmaceutical University, Guangdong Province, China), kept them under SPF conditions, provided them with irradiated food and autoclaved water, and maintained 12 h light/dark cycles in the center. The mice were randomly separated into groups of five (one group per cage). No animals got sick or died prior to the experimental endpoint. The mice were euthanized by cervical dislocation according to the Animal Management Rules of the Ministry of Health of the People’s Republic of China (Document no. 55, 2001). All experiments were approved and performed in compliance with the guidelines of the First Affiliated Hospital of Guangdong Pharmaceutical University Animal Experimentation Ethics Committee (Ethics Approval Number: FAHGPU20160316).

### Cell line, cell culture, peptide synthesis and radionuclide ^131^I

The human CAL-62 cell line was kindly provided by the Stem Cell Bank, Chinese Academy of Sciences, and cultured following the protocols in the product sheets [28]. Briefly, CAL-62 cells were maintained in complete DMEM (Gibco, USA) supplemented with 10% heat inactivated fetal bovine serum (FBS), 100 U of penicillin/mL and 100 μg of streptomycin/mL and were cultured at 37 °C with 5% CO_2_.

The Nthy-ori 3-1 cell line was generous gifted by Professor Libo Chen (The Sixth People's Hospital Affiliated to Shanghai Jiaotong University, P.R. China). Nthy-ori 3-1 cells were cultured in RPMI 1640 medium (Gibco) with 10% FBS in a 37 °C incubator with 5% CO_2_ [29].

Caerin 1.1 (GLLSVLGSVAKHVLPHVLPHVVPVIAEHL-NH_2_, simplified henceforth as F1) and the control peptide (GTELPSPPSVWFEAEF, simplified henceforth as P3) as a negative control were synthesized by China Peptides Proprietary Limited (Shanghai, China). The purity of the peptides was determined to be greater than 95% by reverse-phase HPLC at Mimotopes.

Radionuclide ^131^I was purchased from HTA Co., Ltd. (Beijing, China).

### MTT assay

Cell proliferation was determined by MTT assay (Sigma, St. Louis, MO, USA) following manufacturer’s instructions. Briefly, CAL-62 or Nthy-ori 3-1 cells were cultured separately in flat-bottomed 96-well plates. Different concentrations of F1 (0, 1, 5, 10, 15, and 20 μg/mL) and P3 (0, 1, 5, 10, 15, and 20 μg/mL) were added to 5 × 10^3^ CAL-62 or Nthy-ori 3-1 cells and cultured overnight at 37 °C with 5% CO_2_. Each treatment was performed in triplicate. Then, 10 μL MTT reagent was added, and the cells were cultured for another 4 h. Then, 150 μL DMSO solution (Sigma, USA) was added, and the plate was left at room temperature in the dark for 2 h. The absorbance at 570 nm were analyzed with an ELISA plate reader (Multiskan GO, Thermo Fisher Scientific) according to the manufacturer’s protocol. The IC_50_ value and the survival percentage (S%) were calculated for each peptide using the method described previously [30].

### Plate colony formation assay

For the plate colony formation assay, CAL-62 cells were plated into 6-well plates at a density of 800/well, and different concentrations of F1 (0, 1, 8, 10, and 15 μg/mL) were added. The cells were cultured for 7 days and then fixed in 4% paraformaldehyde and stained with crystal violet staining solution (Beyotime, China). The cell colonies were counted and analyzed by Image-Pro Plus Analysis software.

### Cell wound scratch assay

A wound scratch assay was used to examine the migratory ability of CAL-62 cells. Cells were seeded into 6-well plates (10^6^ cells/well). When the cells reached confluence as a monolayer, a 10 μL pipette tip was used to gently scratch a line across the center of the monolayer of each well. Then, the cells were treated with different concentrations of F1 (0, 5, 10, 15, and 20 μg/mL) for 24 h. The wells were washed with warm PBS to remove detached cells, replenished with complete medium, and cultured for 24 h at 37 °C with 5% CO_2_. Images of each scratch were taken at 0, 6, 12 and 24 h under an inverted microscope (Olympus, Japan; IX73). We used Image-Pro Plus Analysis software to measure the migration distance of cells, and the average migration distance of three cells was used to represent the wound-healing capacity. The wound-healing percentage (S%) was calculated using the following equation:

$$S\% = \, \left( {{\text{the initial scratch width }} - {\text{ the current scratch width}}} \right)/{\text{the initial scratch width }} \times \, 100\% .$$

### Cell cycle analysis

For the cell cycle analysis, 5 × 10^5^ CAL-62 cells were seeded into 6-well plates and treated with different concentrations of F1 (0, 10, 20, 30, and 40 μg/mL) for 24 h and then collected and fixed in 75% ethanol at 4 °C overnight. The cells were incubated with PI/RNase Staining Buffer (BD Pharmingen, San Diego, CA, USA, 550825) at 37 °C for 30 min. The phase distribution of the cell cycle was assessed by a flow cytometer (Novocyte D2060R, Agilent, USA) equipped with NovoExpress software.

### Western blotting

Total protein was extracted by a previously described method [16]. Briefly, the protein concentration of F1-treated CAL-62 cells was detected by the BCA method (Thermo Scientific, USA), and the extracts containing equal amounts of proteins (20 μg) were electrophoresed in the desired concentration of polyacrylamide gel according to the size of the target protein and then transferred to a PVDF membrane. The membrane was blocked for 1 h with 5% bovine serum albumin (BSA) in TBS-Tween 20 buffer and incubated with different rabbit monoclonal antibodies (1:1000 dilution; p-AKT, CST #4060S; AKT, CST #4691) and mouse monoclonal antibodies (β-actin, Proteintech, 66009–1-Ig). The membrane was then incubated with anti-rabbit/mouse antibody (1:40,000 dilution; goat anti-rabbit IgG (H + L) HRP, Bioworld, BS13278; goat anti-mouse IgG (H + L) HRP, Bioworld, BS12478) at room temperature for 1 h, and ECL detection reagents (Merck Millipore, USA) were used to visualize the membrane with an image scanner Protein Simple (Santa Clara, CA, United States). Blots were analyzed by Image-Pro Plus Analysis software (Media Cybernetic, Rockville, Maryland, USA).

### Preparation of ^131^I-labeled F1 (^131^I-F1)

First, 10 mg chloramine-T trihydrate (Sigma, USA) was mixed with 10 ml of distilled water and stored at room temperature in the dark. Then, 40 μg of F1 was transferred to a 1-ml sterile EP tube; next, we added 100 μl of Na–^131^I solution (1 mCi) and 100 μl of freshly prepared chloramine-T solution. We used a vortex mixer (Scientific Industries, USA, Vortex-Genie 2 T) to vibrate and mix the contents for 10 min at room temperature. Next, we used paper chromatography to evaluate the labeling rate of ^131^I-F1 [28], and its radioactivity was measured by a γ counter. Paper chromatography was also applicable to ^131^I. The γ counting curve of them were drawn using GraphPad Prism 5.0 and the labeling rate was obtained by calculating the area under the curve.

$$R_{{\text{f}}} {\text{ = distance from the}}^{131} {\text{I or}}^{131} {\text{I}} - {\text{F}}1{\text{ to the}}\;{\text{origin}}/{\text{distance from the saline front to the origin}}$$

$$\begin{aligned} {\text{Labeling rate }}\left( \% \right) & = {\text{radioactivity peak area integral of}}^{131} {\text{I}} \\ & - {\text{F1}}/{\text{total radioactivity peak area integral}}{.} \\ \end{aligned}$$

### Tumor formation assays in the animal model

CAL-62 cells (5 × 105) in 100 μl PBS were inoculated subcutaneously into nude mice to establish a subcutaneous xenotransplantation model. When the tumor diameters were close to 6 mm, the mice were used for the following experiments. The tumor diameter was calculated using a method described elsewhere [30]. The tumor sizes were monitored every 3 days using a digital caliper (Mitutoyo, Japan, CD-15APX), and the tumor volume was calculated according to the following equation: ${\text{volume }} = {\text{ width}}^{2} \times {\text{length}}/2$.

### ^131^I-F1 treatment experiment

To reduce the unnecessary thyroid uptake of ^131^I, before the in vivo experiments, all nude mice were fed potassium iodide in a concentration of 0.1% for 3 days to saturate the thyroid. CAL-62 tumor-bearing mice were randomly divided into four groups (*n* = 5 per group) that received one of the following treatments: PBS, F1 only, ^131^I-F1, ^131^I only. The mice were injected intratumorally with 100 µl PBS containing 8 µg of F1 peptide, 200 µCi of ^131^I or 8 µg of F1 peptide labeled with 200 µCi ^131^I every 3 days for a total of three times. Then, 3 days after the final treatment, the mice were sacrificed, and the tumors were isolated and weighed.

### Hematoxylin and eosin (H&E) and TUNEL staining

The tumor tissues were then dissected, dehydrated, and embedded, and H&E and TUNEL staining were used to detect apoptosis. The sections were scored independently by two pathologists. The score was based on both the proportion of positively stained cells and the intensity stained tumor cells.

### Statistical analysis

All experiments were replicated at least three times. The MTT data were analyzed by *t* test using Prism 5.0 (GraphPad Software, San Diego, CA, USA), and the remaining experiments were analyzed by ANOVA. Differences were considered significant at *P* < 0.05.

## Results

### F1 inhibited the proliferation of CAL-62 cells but was not cytotoxic to normal cells at the same concentration

**Fig. 1:**
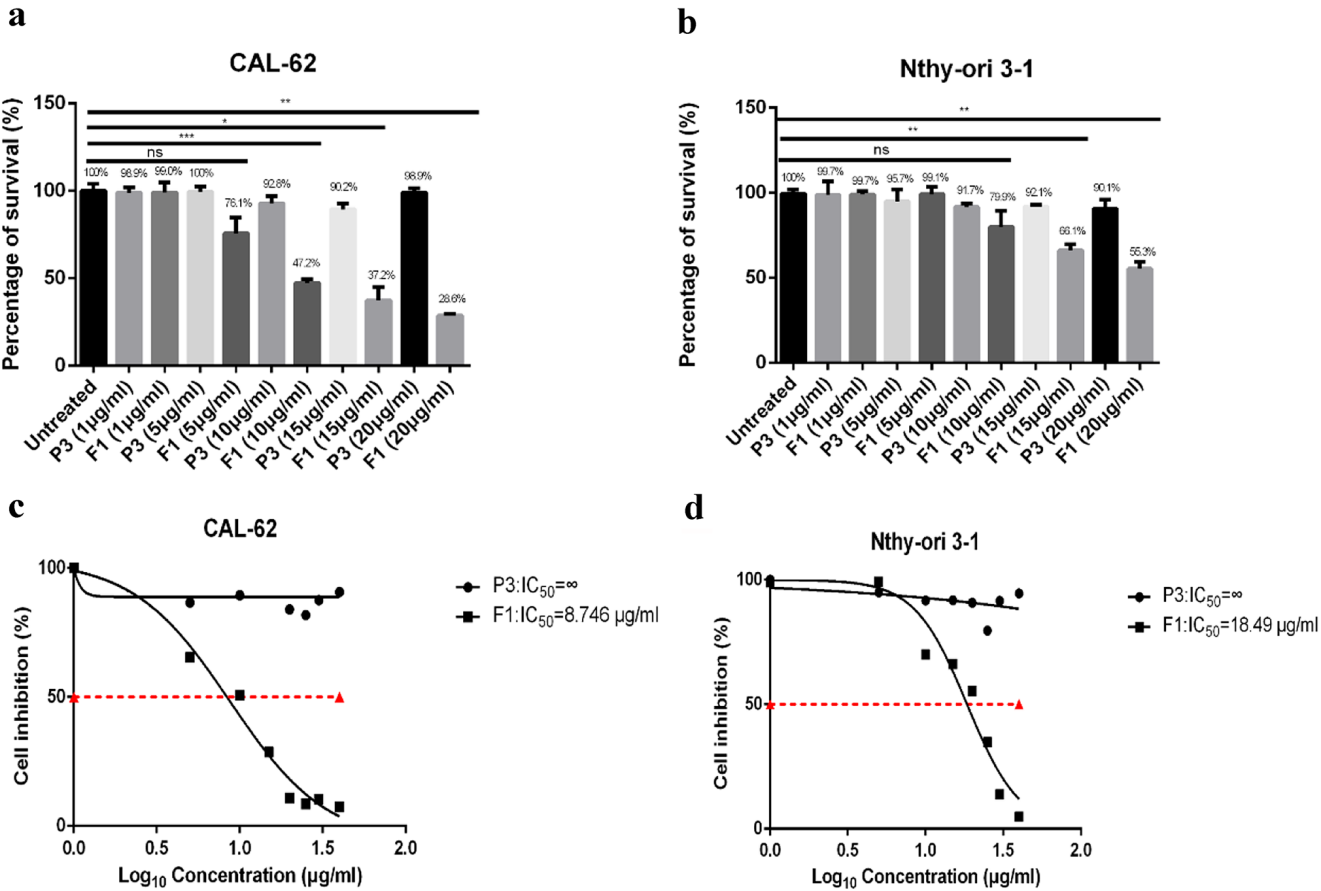
F1 inhibited the proliferation of the cells according to the MTT assay. A total of 5 × 10^3^ CAL-62 cells (**a**) and Nthy-ori 3-1 cells (**b**) were cultured independently in media with different concentrations of F1 or P3 for 24 h before the MTT assay was performed. Moreover, the IC_50_ values of F1 were determined. For this, 5 × 10^3^ CAL-62 cells (**c**) and Nthy-ori 3-1 cells (**d**) were incubated in culture medium alone or in medium with different concentrations (1, 5, 10, 15, 20, 30, 40 and 50 µg/mL) of F1 or P3 peptide overnight before the MTT assay was performed. Each bar represents the means of three replicates, and the error bars represent the standard deviations. The data are representative of two independent experiments. A two-tailed *t* test was used to calculate the *P* values: *P < 0.05; **P < 0.01; ns, not significant

For the MTT assays, human normal thyroid tissue cells (Nthy-ori 3-1) and anaplastic thyroid carcinoma cells (CAL-62) were used to investigate the effects of F1 and the control peptide (P3) (Fig. 1), and untreated and P3-treated cells were used as negative controls, as described elsewhere [17]. The F1 peptide showed no significant activity at 1 μg/mL. When the concentration of F1 reached 5 μg/mL and 10 µg/mL, the F1 peptide inhibited CAL-62 cell growth but had no cytotoxic effects on Nthy-ori 3-1 cells. As shown, for CAL-62 cells, the cell proliferation rates were 76.1% and 47.2% at 5 μg/mL and 10 µg/mL, respectively, while the Nthy-ori 3-1 cell survival rates were 99.1% and 79.9%, respectively (Fig. 1a, b). In addition, at a concentration of 15 µg/mL, F1 remarkably inhibited the proliferation of both cell lines, and only 37.2% of CAL-62 cells and 66.1% of Nthy-ori 3-1 cells remained viable. At 20 µg/mL, the CAL-62 and Nthy-ori 3-1 cell survival rates were 28.8% and 55.3%, respectively. Therefore, when the concentration of F1 peptide was less than 15 µg/mL, it had no cytotoxic effect on normal cells, which was consistent with the results of previous studies [15, 30]. Next, we determined the half-maximal inhibitory concentration (IC_50_) values for the F1 peptide in CAL-62 and Nthy-ori 3-1 cells; the IC_50_ values were 8.746 µg/mL and 18.49 µg/mL, respectively (Fig. 1c, d). Thus, only the F1 peptide significantly inhibited the growth of human cancer cell lines in a dose-dependent manner, and P3 was not active. In addition, at a high concentration of 10 µg/ml, F1 showed relatively low cytotoxicity to normal cells and significantly inhibited cancer cell proliferation.

### F1 suppressed the growth and migration of CAL-62 cells in vitro

**Fig. 2:**
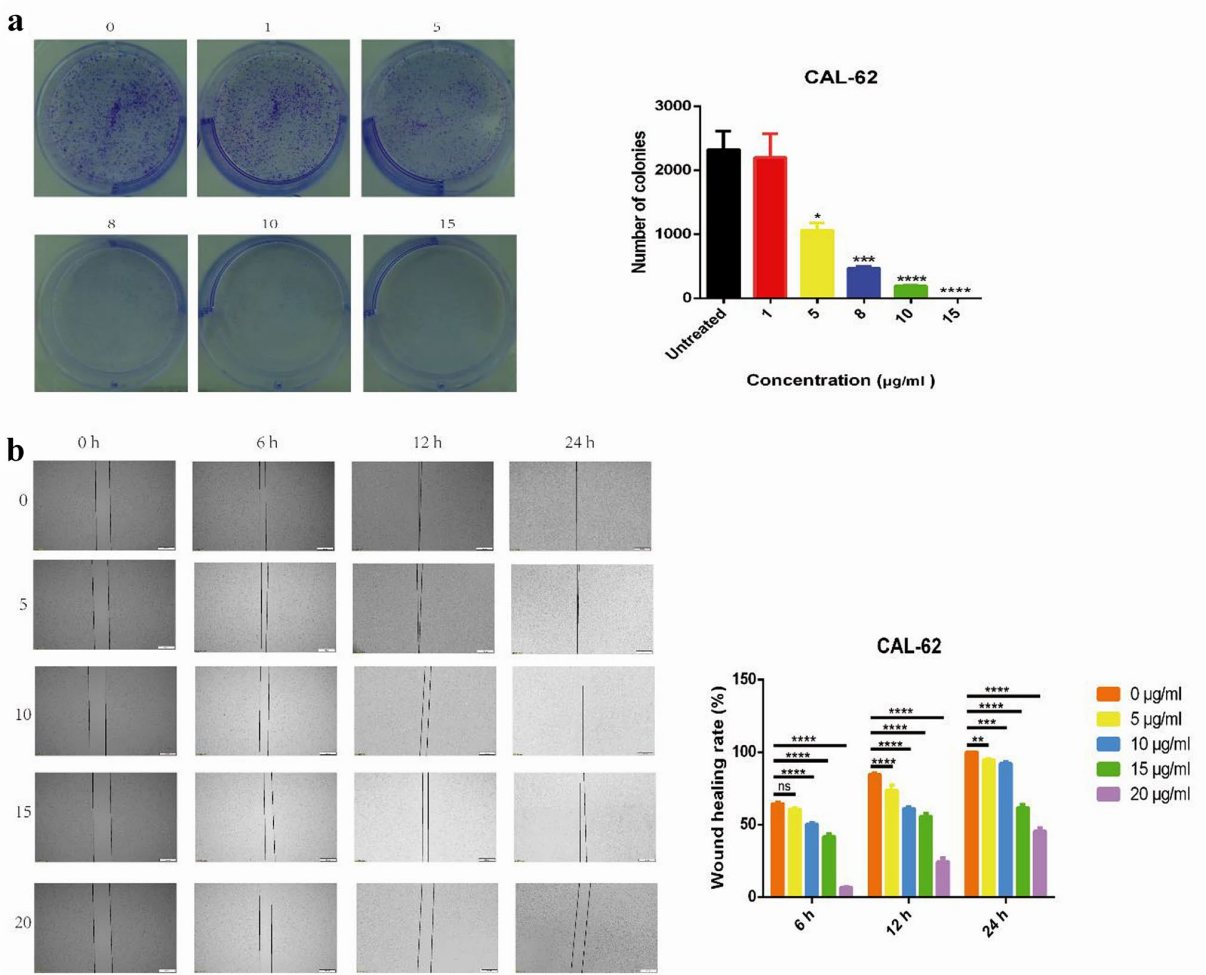
Proliferation and migration of CAL-62 cells were suppressed by F1. **a** Colony forming assay was used to determine the cell proliferation of CAL-62 cells treated with different concentrations of F1 (0, 1, 8, 10 and 15 µg/mL). The number of colonies per well was counted with Image-Pro Plus Analysis software. Representative images of the colony formation assay in the indicated cells (left); statistical analysis of the colony formation results (right). ANOVA was used to calculate the *P* values: *P < 0.05, ***P < 0.001, ****P < 0.0001. **b** Scratch wound healing assay was performed to determine the cell migratory ability of CAL-62 cells treated with different concentrations of F1 (0, 5, 10, 15 and 20 µg/mL). Scale bar: 500 μm. Images of the wound healing assay showing different migratory capacities at 4 regular time intervals (left); statistical analysis of the wound healing assay results (right). ANOVA was used to calculate the *P* values: **P < 0.01; ***P < 0.001; ****P < 0.0001; ns, not significant

To investigate whether F1 affected the biological behavior of CAL-62 cells, plate colony formation and cell wound scratch assays were performed to evaluate cell growth and migration in vitro. As expected, the plate colony formation assays showed that in CAL-62 cells, F1 inhibited cell proliferation (*P* < 0.05; Fig. 2a); F1 remarkably suppressed the migration capacity of CAL-62 cells (Fig. 2b). In the F1 treatment groups, tumor cells grew slower than those in the control group according to the wound closure at 24 h (*P* < 0.05).

### F1 treatment may induced CAL-62 cell apoptosis by arresting cells in the S phase of the cell cycle

**Fig. 3:**
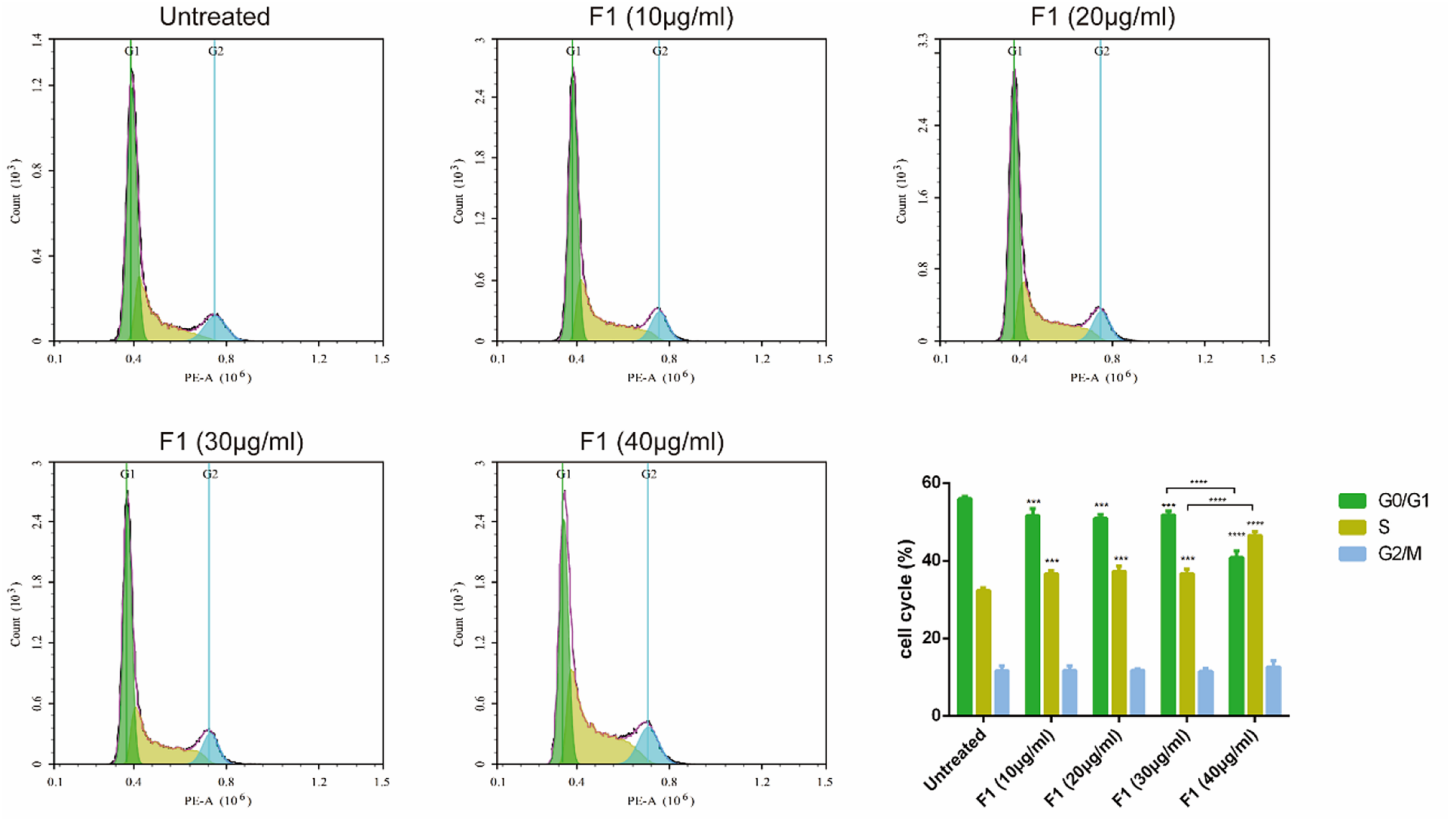
F1 treatment induced CAL-62 cell apoptosis and S-phase cell cycle arrest. A total of 1 × 10^6^ CAL-62 cells were treated with different concentrations (0, 10, 20, 30, and 40 µg/mL) of F1 for 24 h, and the cells were collected for FACS analysis (*n* = 3). ANOVA was used to calculate the *P* values: *P < 0.05, **P < 0.01, ***P < 0.001, ****P < 0.0001

Next, we investigated whether apoptosis of CAL-62 cells was responsible for the cell cycle effects induced by F1 treatment. For this purpose, we treated CAL-62 cells with different concentrations (0, 10, 20, 30, and 40 µg/mL) of F1 for 24 h. The results showed that when the concentration of F1 increased, compared with the 0 µg/mL group, the G1 phase decreased and the S phase increased, with significant statistical difference (*P* < 0.001). Compared with the 30 µg/mL group, the G1 phase of the 40 µg/mL group was significantly decreased (*P* < 0.0001), increased significantly in S phase (*P* < 0.0001). Notably, it was previously reported that the apoptosis of tumor cells is induced by caerin peptides [17]. Thus, we investigated whether the programmed death of CAL-62 cells plays a key role in the cell cycle. Therefore, F1 might induce CAL-62 cell cycle arrest at the S phase and exist in a concentration dependence (Fig. 3).

### F1 may inhibit proliferation of CAL-62 cells by inhibiting phosphorylation of Akt

**Fig. 4:**
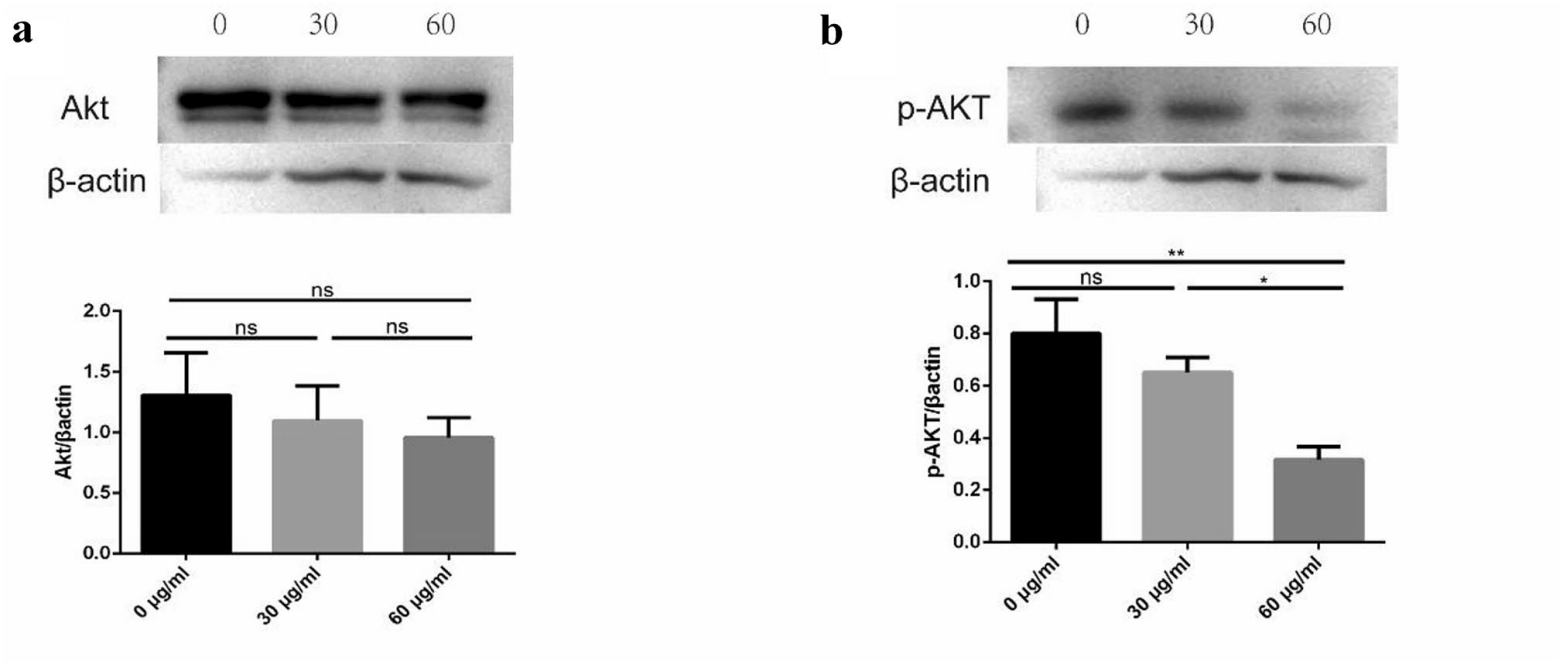
Effects of F1 on AKT and p-AKT protein expression were analyzed by western blotting. CAL-62 cells were treated with different concentrations of F1 for 24 h. One representative western blotting result of β-actin expression was used as a control. AKT (**a**), p-AKT (**b**). ANOVA was used to calculate the *P* values: *P < 0.05, **P < 0.01

Western blotting was used to further detect the possible antitumor mechanisms of F1 on CAL-62 cells, and then the signaling pathways were analyzed by immunoblotting. Compared with the control treatment, treatment with different concentrations of F1 (0, 30, and 60 μg/mL) decreased the expression of p-AKT, while AKT did not change significantly (Fig. 4). These results indicated that the F1 may inhibit proliferation of CAL-62 cells by inhibiting phosphorylation of Akt.

### Labeling rate of ^131^I-F1

**Fig. 5:**
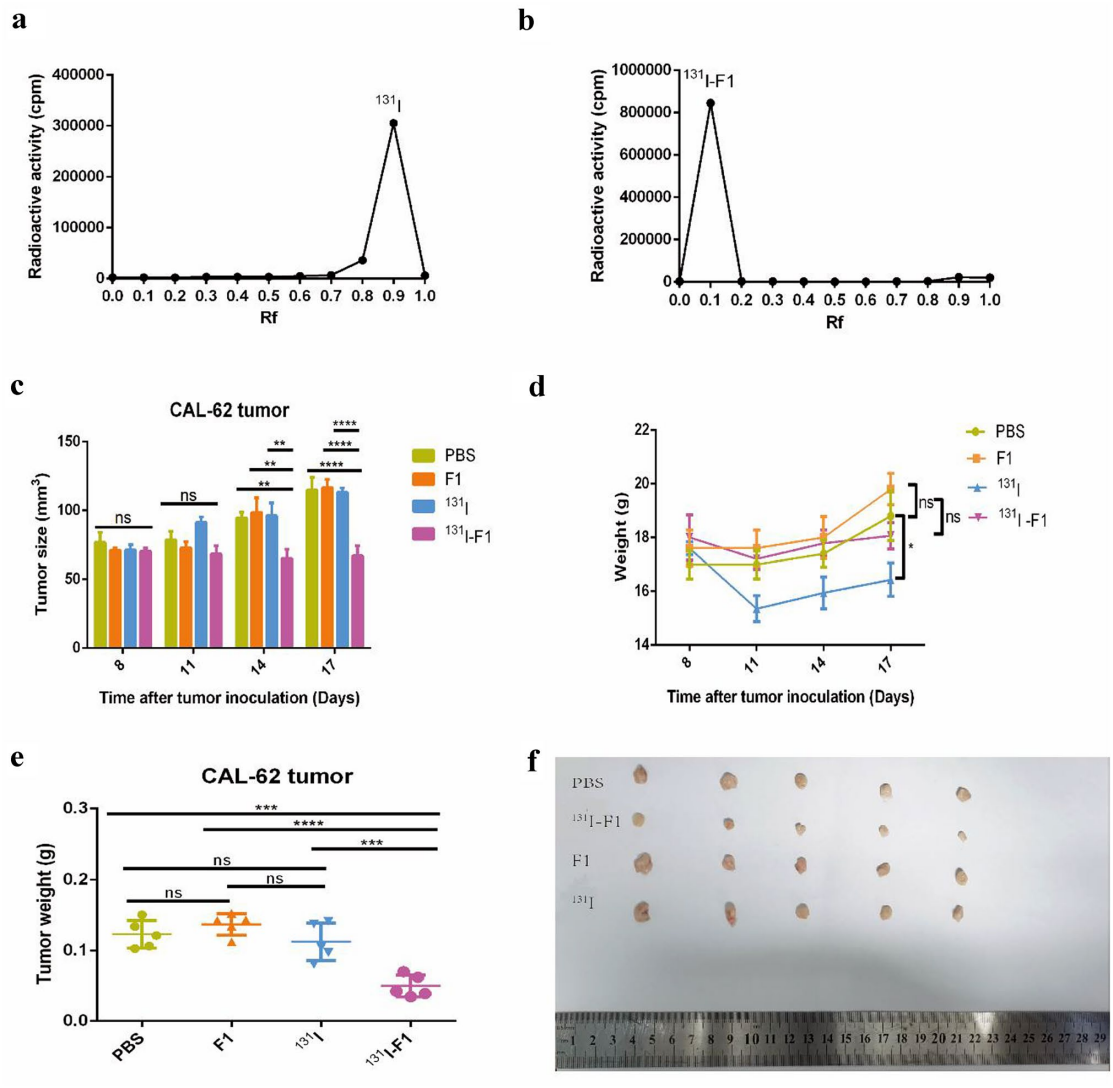
Inhibition of ^131^I-F1 affected CAL-62 cell proliferation in vivo. **a** ^131^I was located at *R*_f_ of 0.8–1.0 and the γ counting curve of ^131^I was drawn. **b** ^131^I-F1 was located at *R*_f_ of 0–0.2, the γ counting curve of ^131^I-F1 was drawn and the labeling rate was calculated to be approximately 95% ± 2%. **c** Growth in tumor size of CAL-62 tumor-bearing nude mice with different treatments (*n* = 5). **d** Curves for weight change among the mice in different treatment groups. **e** Tumors were isolated from individual mice and analyzed for weight. **f** Photo of isolating tumors in different treatment groups. The error bars represent the mean ± SD from three independent experiments. *P < 0.05, **P < 0.01, ***P < 0.001, ****P < 0.0001

We developed an ^131^I-F1 RIC using the chloramine-T method. One microliter of ^131^I-F1 sample was spotted on a 1 × 12 cm strip of chromatography paper as the stationary phase and was placed on a class tube and developed with normal saline as the mobile phase. After the mobile phase, the paper was cut off every 1 cm, and the radioactivity was measured by a γ counter. Chromatography paper was also applicable to ^131^I. The results showed that ^131^I was located at *R*_f_ of 0.8–1.0 (Fig. 5a), and when ^131^I was used to label F1, the position of high radioactive activity had changed to *R*_f_ of 0–0.2 (Fig. 5b). The γ counting curve of them were drawn (Fig. 5). The final radiolabeling rate was calculated to be 95% ± 2% (Fig. 5b).

### ^131^I-F1 affected CAL-62 cell proliferation in vivo

Next, we studied whether F1 was able to inhibit CAL-62 cell growth in vivo. Nude mice were subcutaneously injected with 5 × 10^5^ CAL-62 cells. When the tumor diameters were close to 6 mm, the mice were treated with 100 µl PBS containing 8 µg of F1 peptide, 200 µCi of ^131^I or 8 µg of F1 peptide labeled with 200 µCi ^131^I every 3 days for a total of three times. Consistent with the in vitro findings, tumors treated with ^131^I-F1 treatment grew slower than those treated with the control (Fig. 5c). Three days after the final treatment, the weights of the mice increased to 18.80 ± 2.049 g (PBS), 19.80 ± 1.304 g (F1) and 18.06 ± 1.078 g (^131^I-F1) times higher than those prior to treatment, whereas those of the ^131^I group decreased 16.43 ± 1.386 g (Fig. 5d). The mice were euthanized, and the tumors were isolated and weighed. At the end of the experiment, the weights of tumors in the ^131^I-F1 group were significantly reduced compared to those in other groups (Fig. 5e).

**Fig. 6:**
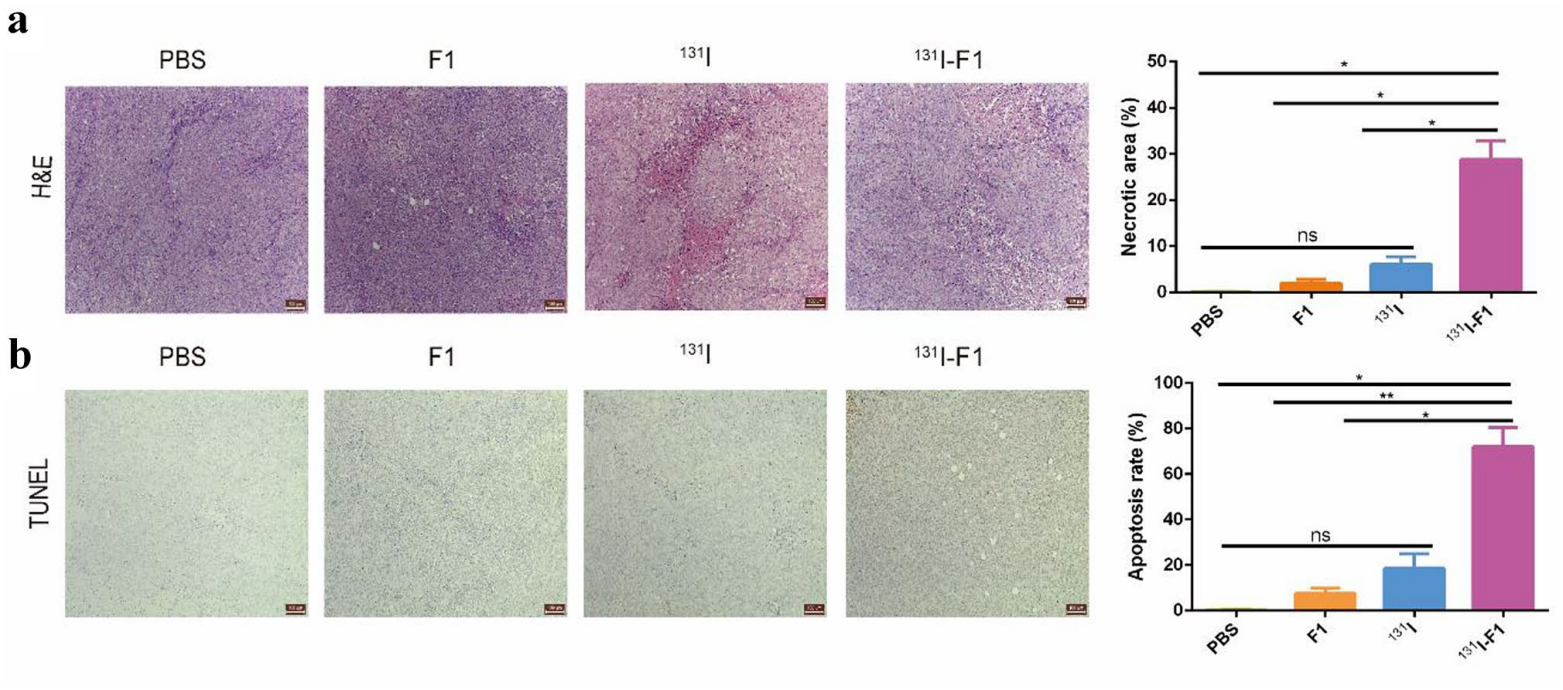
Tumor inhibition by ^131^I-F1 in vivo. H&E (**a**) and TUNEL (**b**) staining of tumors with different treatments. Scale bar: 100 μm. The error bars represent the mean ± SD from three independent experiments. *P < 0.05, **P < 0.01

H&E-stained tumors with different treatments are presented in Fig. 6a. Compared to the normal cell morphology in the PBS group, the ^131^I-F1 group exhibited the most apparent ablation of tumor cells (*P* < 0.01), while only a few necrosis events occurred in the ^131^I and F1 groups (*P* > 0.05). The apoptosis study (TUNEL staining) is illustrated in Fig. 6b. The ^131^I-F1 group had higher apoptosis signals than the other groups (P < 0.05). The findings of H&E and TUNEL staining were basically consistent and were in accordance with the antitumor effects observed with different treatments in vivo.

## Discussion

ATC is one of the most aggressive and deadliest human malignancies [31, 32], it is rare, as less than 2% of all thyroid carcinomas (TCs), but ATC accounts for 14–39% of TC-related deaths [33, 34]. Patients with ATC usually show rapidly growth of cervical masses, regional cervical lymph nodes are often involved, and about half of the patients have distant metastasis [33]. The median survival of ATC remains approximately 3–5 months, and the 1-year survival rate is approximately 20%; most patients die from the rapid onset of clinical symptoms leading to asphyxiation [35, 36]. According to the guidelines of American Thyroid Association (ATA), the first-line treatment of ATC includes surgical resection and external radiotherapy [37, 38]. Although total thyroidectomy and high-dose radiotherapy can improve the survival rate, no therapies are available to cure or to prolong the survival of patients with ATC [39], so multimodal therapy (surgery, external beam radiation therapy and chemotherapy) and new therapies are urgently need.

All of these data indicate that the combination of RT and immunotherapy may be beneficial in the treatment of ATC. F1 immunotherapy is effective for tumor cells. Gene hyperactivation, overexpression, or mutation and dysregulated signaling are the characteristics of many cancer cells [40]; these alternations activate many signaling proteins and stimulate the activation of many signaling pathways, including PI3K/Akt. PI3K is activated through direct interaction with the p85α subunit or via activated Ras, and a key downstream mediator of the PI3K signaling cascade is the serine/threonine (Ser/Thr) kinase Akt. The ErbB receptor activates PI3K to stimulate Akt activity and protects cells from apoptosis [41]. Additionally, increased activity of the PI3K/AKT pathway has been observed in a large number of malignancies [42]. Therefore, one possible mechanism of the effects of immunotherapy on tumor cells is that F1 regulates the function of cells, resulting in the inactivation of the PI3K/Akt pathway and suppression of its overexpressed or mutated signaling components, which promotes cell apoptosis. There have been publications showing that the TNF-α pathway plays important roles in regulating tumor proliferation, migration, invasion and angiogenesis, and the host-defense peptides caerin 1.1 and 1.9 stimulate TNF-α-dependent apoptotic signals in human cervical cancer (HeLa) cells [16]. The mechanism of F1 in the treatment of ATC has never been explored. In our study, the results (Figs. 1 and 2) revealed that the inhibitory effect of F1 on CAL-62 cell growth occurred via cell apoptosis in a dose-dependent manner without cytotoxicity to normal cells at the same concentration. Next, the results (Fig. 4) confirmed that the protein expression levels of p-AKT in CAL-62 cells treated with F1 were significantly decreased, indicating that the anti-tumor activity of F1 might be related to the inhibition of phosphorylation of Akt.

The most basic biological characteristic of malignant tumors is the unlimited proliferation of cancer cells, and dysregulation of the cell cycle is the biological basis for uncontrolled cell proliferation [43]. Thus, the important events in tumor cell growth, such as cell cycle transition, growth factor production or cell death, are controlled by transcriptional regulation [44, 45]. Studies have shown that Deregulation of the mammalian TOR (mTOR) signaling network is associated with cancer, because MTOR is activated in a variety of cellular processes, such as tumorigenesis, angiogenesis, and lymphocyte activation, and is dysregulated in a variety of cancers [46–48]. Biozentrum etc. investigated CAD (carbamoyl-phosphate synthetase 2, aspartate transcarbamylase, and dihydroorotase) as a potential mTORC1 effector. CAD played a role in pyrimidine synthesis, which was a conserved metabolic pathway necessary for S phase progression [49]. In addition, S phase was an important stage of cell synthesis [50]. Cell cycle progression is regulated by multiple overlapping checkpoints, which are blocking S phase (G1–S checkpoint), the progression through S phase (intra S phase checkpoint), the initiation of mitosis (G2 M checkpoint) and the initiation of anaphase (spindle checkpoint). These checkpoints block cell cycle in response to DNA damage or other cell damage [51]. In our research, we found that F1 has antitumor effects on the metastasis and invasion of CAL-62 cells (Fig. 2), which may be involved in the regulation of the cell cycle. Compared to the untreated control cells, F1-treated cells exhibited S phase arrest, which may occur in a concentration-dependent manner. This may have reduced DNA replication, delayed or prevented S phase entry, inhibited DNA synthesis, or induced DNA fragmentation (Fig. 3) [48, 52].

Next, according to the principle of RIT, including mAb combination with cytotoxic drugs, conjugation with radionuclide’s and immunological effector cells [53, 54], we prepared ^131^I-F1 and proved it was able to inhibit CAL-62 cell proliferation in nude mice. First, ^131^I-F1 was successfully synthesized using the chloramine-T method. The results of the cell proliferation test showed that there was a significant difference in the ^131^I-labeled F1 group versus the ^131^I group in vitro [28]. Furthermore, a subcutaneous xenograft model was used to investigate the antitumor activity of ^131^I-F1 in vivo. Both the tumor size and the weight of the ^131^I-F1 group were significantly decreased compared with those of the control groups, with no signs of weight loss or death (Fig. 5c–e). The effect of ^131^I-F1 on CAL-62 cell proliferation was verified by H&E and TUNEL staining (Fig. 6a, b). We measured the labeling rate of 1mci ^131^I with different grams of F1, our results confirmed that the labeling rate was the highest when F1 was equal to 40 μg. According previous stud [55, 56], 200 μCi ^131^I was used in our study, which contains 8 μg of F1. The MTT result indicated that 5 μg of F1 begin to inhibit the growth of CAL-62 cell in vitro (Fig. 1); however, our previous experiment indicated that 30 μg of F1 and F3 had a significant inhibitory effect on cervical cancer cell (TC-1) in vivo [15]. Therefore, 8 μg of F1 is unlikely to inhibit CAL-62 cell in our study. Indeed, 8 μg of F1 cannot inhibit CAL-62 cell growth, ^131^I significantly improved the therapeutic efficiency of F1.

Interestingly, in an in vivo study, we found that the body weight of mice in the ^131^I treatment group decreased over time, but not the ^131^I-F1 group or F1 group. We considered that the possible reason was that ^131^I-F1 or F1 was confined to the tumor after tumor injection, but ^131^I might enter the microcirculation through the blood vessels in the tumor, and then enter the circulatory system in the body. The radiation which entered the body may cause acute injuries, similar to Acute Radiation Sickness or Syndrome (ARS), characterized by weight loss [57], indicating that ^131^I-F1 was safer and had smaller side effects. We will explore the mechanism of ^131^I-F1 for ATC in vivo and try to further uncover this reason.

It is more common in targeted radioimmunotherapy to inject radiopharmaceuticals into intravenously, we will investigate the therapeutic effect of I^131^-F1 on ATC by intravenous injection, and explore the SPET imaging and biodistribution in vivo. We did not perform the in vivo stability of ^131^ I-F1 and the biodistribution to major organs in our current study. However, our previous study of ^125^I-caerin 1.9 (^125^I-F3) demonstrated that SPECT imaging and biodistribution in a breast cancer model in vivo [58]. ^131^I-F1 may has similar stability and biological distribution, and we are currently attempting to explore the ^131^I-F1 biodistribution, and the efficacy between ^131^I-F1 and ^131^I-F3 against thyroid cancer using our current method.

## Conclusions

In summary, our study demonstrates for the first time that ^131^I-F1 can inhibit CAL-62 tumor growth and migration. We also found that F1 arrested cells in the S phase to induce apoptosis and inhibited tumor growth to inhibit phosphorylation of Akt. At present, we are studying whether ^131^I-F1 is suitable for the treatment of ATC in situ, which is still a prominent problem worldwide.
